# High urinary interleukin-2 in late post-transplant period portends a risk of decline in kidney allograft function: a preliminary study

**DOI:** 10.1186/s13104-017-2936-7

**Published:** 2017-11-21

**Authors:** Andriy V. Trailin, Marina V. Pleten, Tetyana I. Ostapenko, Nadiia F. Iefimenko, Olexandr S. Nykonenko

**Affiliations:** 1 Department of Laboratory Diagnostics and General Pathology, State Institution “Zaporizhzhia Medical Academy of Postgraduate Education Ministry of Health of Ukraine”, 20 Winter boulevard, Zaporizhzhia, 69096 Ukraine; 2 Department of Transplantology and Endocrine Surgery with the Course of Cardiovascular Surgery, State Institution “Zaporizhzhia Medical Academy of Postgraduate Education Ministry of Health of Ukraine”, Zaporizhzhia Regional Hospital, 10 Orikhiv highway, Zaporizhzhia, 69050 Ukraine

**Keywords:** Renal transplantation, Serum, Urine, Interleukins, GFR

## Abstract

**Background:**

Predictive factors for the rate of decline in kidney allograft function beyond the first post-transplant year have not been thoroughly studied. We aimed to determine whether a single measurement of serum and urinary interleukin 2, interleukin 8 and interleukin 10 at 1–15 years after kidney transplantation could predict a decline in estimated glomerular filtration rate (eGFR) over a 2-year period.

**Results:**

Greater serum concentrations of interleukin 8 and interleukin 10 in 30 recipients of kidney allograft at enrollment were associated with lower eGFR after 1 year (beta = − 0.616, p = 0.002 and beta = − 0.393, p = 0.035, respectively), whereas serum concentrations of interleukin 8 also demonstrated significant association with eGFR after 2 years of follow-up (beta = − 0.594, p = 0.003). Higher urinary interleukin 2 concentrations were associated with lower eGFR at baseline (rho = − 0.368, p = 0.049) and after the first (beta = − 0.481, p = 0.008) and the second year (beta = − 0.502, p = 0.006) of follow-up. Higher urinary interleukin 2 concentrations predicted certain decline in eGFR of ≥ 25% from baseline after 1 year of follow-up in logistic regression: odds ratio = 2.94, confidence interval 1.06–8.18, p = 0.038. When combined with time after transplantation, urinary interleukin 2 demonstrated good accuracy in predicting rapid decline in eGFR by > −5 mL/min/1.73 m^2^/year (area under the receiver-operator characteristic curve: 0.855, confidence interval 0.687–1.000, and p = 0.008).

**Conclusions:**

Our findings suggest that urinary interleukin 2 in the late period after kidney transplantation has promise in identifying patients who are at risk for progressive loss of graft function in a short-time perspective and need closer monitoring.

## Background

Kidney transplantation is a treatment of choice for end-stage renal disease [1]. At present, in spite of excellent 1-year renal graft survival, an increase in long-term survival rate remains an unresolved issue. Thus, preservation of allograft function in the late period after transplantation and prevention of chronic allograft dysfunction have become the main challenge [1]. Risk stratification for progressive decline in graft function is a prerequisite for developing strategies for saving graft function and improving its survival [2]. However, predictive factors for the rate of decline in kidney function beyond the first post-transplant year have not been thoroughly studied [2].

Core needle biopsy is considered to be a gold standard for the diagnostics of allograft pathology and can also provide prognostic information [3]. However, biopsy is prone to sampling error and also carries certain unavoidable risks. These clinical limitations have inspired a search for noninvasive biomarkers. Such biomarkers for transplanted kidney can help better understand pathogenesis, assess immune risk, detect early injuries, make differential diagnosis, and guide therapeutic decision-making [4].

It is well accepted, that proinflammatory interleukins (IL), as IL-2, and antiinflammatory ones, as IL-10, play a major role in induction and effector phases of all immune and inflammatory responses [5]. IL-8 is a CXC-chemokine, which is produced by leukocytes, endothelial and epithelial cells after stimulation, and participates in inflammatory processes, bacterial and viral infections [5]. Interleukins and chemokines are known to modulate lymphocytes and kidney cells interactions to mediate kidney injury and fibrosis [6]. Many studies have examined the potential of serum IL-2 [7], IL-8 [8, 9] and IL-10 [7, 8, 10, 11] for early assessment of kidney allograft (KAG) state. When measured in urine, IL-2 [12, 13], IL-8 [9, 13–15], and IL-10 [12–14] also allow early diagnostics of KAG insults. However, findings from these studies have often been inconclusive. Contradictory results regarding the relationship between serum and urinary interleukins and KAG function have been reported in the literature [14–16]. The study population was often restricted to the recipients of allograft from living donor [7, 17, 18]. The majority of studies were constrained to early post-transplant period [7, 12, 13, 15], and were focused on the diagnostics of acute tubular necrosis [8, 9, 14, 15], acute rejection [7, 9, 10, 12, 13], or infection [9, 13]. There have been few studies paying attention to the problem of chronic graft injury [11, 16, 18]. Though few papers describe prognostic significance of serum [17] and urinary interleukins [9, 13, 15], they used acute rejection [9, 13], sustained acute renal failure [15], graft loss [17] or serum creatinine at fixed time [18] as an end-points and disregarded the pattern of progression of graft failure. Meanwhile, the investigation of the association between serum and urinary interleukins and a rate of progression of kidney graft failure would be of interest, because the rate of decline in graft function might be an early indicator of allograft injury and strong predictor of its survival [2, 19]. Also, it seems logical that urinary biomarkers compared with serum’s ones can carry more useful information about KAG state, since the urine specimen provides a representative sampling of the entire kidney allograft. However, currently there is no agreement regarding the preferential use of serum or urine samples for measurement of interleukins [9].

In the present study we decided to check up a hypothesis that serum and urinary interleukins (IL-2, IL-8 and IL-10) in the late postoperative period could predict a decline in glomerular filtration rate (GFR) over a follow-up period of 2 years.

## Methods

### Study population

In total, 90 Caucasian patients receiving a kidney allograft in Zaporizhzhia transplantation center between February 1997 and September 2011 and willing to participate were recruited from September till December, 2012. The criteria of inclusion to the study were the following: the adult kidney allograft recipient, male or female, with primary transplantation from related or deceased donor, with allograft survival of at least 1 year, with eGFR not less than 15 mL/min/1.73 m^2^, and available data on the concentration of all of interleukins (IL-2, IL-8 and IL-10) both in serum and urine. The criteria of exclusion were regular dialysis, acute kidney injury, diseases of immune system, solid tumors, clinical signs of acute infections, and incomplete data on interleukins concentration in serum and urine. A total of 30 patients, 16 males and 14 females, aged 16–55, who fulfilled the inclusion criteria, were enrolled in the study. Induction immunosuppression consisted of anti-IL-2 antibodies and was used in recipients transplanted after 2005. All recipients received triple maintenance immunosuppressive therapy consisting of calcineurin-inhibitor (cyclosporine A or tacrolimus), antiproliferative agent (mycophenolate mofetil or azathioprine), and steroid. All participants gave their informed written consent. This study was approved by the Ethical Review Board of State Institution “Zaporizhzhia Medical Academy of Postgraduate Education Ministry of Health of Ukraine” and carried out in accordance with the ethical standards laid down in the Declaration of Helsinki and the Declaration of Istanbul.

### Laboratory methods

Serum was obtained from the venous blood taken in the morning from patients in a fasting condition. Simultaneously, freshly voided morning urine samples were collected and centrifuged at 3000 rpm for 15 min. We measured serum concentration of urea and creatinine by the urease and Jaffe methods, respectively. We measured urine protein level by the pyrogallol red-molybdate method and urine creatinine concentration by the Jaffe method. For measuring of IL-2, IL-8 and IL-10 aliquots of serum and urine supernatant were frozen and stored at minus 40 °C. Levels of IL-2, IL-8 and IL-10 were determined by enzyme-linked immunosorbent assay (all kits supplied by Vector-Best, Russia) according to the manufacturer’s protocol. The absorbance was measured by using a plate reader «Tecan» Sunrise (Austria). To correct for variations in urine flow the results of total protein, and interleukins in urine spot were normalized to urinary creatinine. As a threshold of normalized proteinuria we considered the level of 15 mg/mmol [19]. For GFR estimation we used a four-variable equation derived from the modification of diet in renal disease (MDRD) study.

### Risk factors and outcomes examined

In this study we assessed a prospective value of a cross sectional measurement of serum and urinary IL-2, IL-8 and IL-10. Archival case records and out-patient cards were used to obtain more information on major risk factors and evolution of allograft function. We collected data on recipient’s age, gender and presence of chronic arterial hypertension, defined as a regular intake of antihypertensive drugs. We also obtained the information related to transplantation: donor source, type of immunosuppression, initial graft function, acute rejection episodes, and time after transplantation. The arterial pressure was measured at enrollment in a sitting position after a 10-min rest period.

For linear regression analysis the initial allograft function was classified as follows: immediate function (0 points), slow function, that is, serum creatinine reduction from transplantation to day 7 < 70% (1 point), delayed function, that is, need for at least one time dialysis in the first 7 days after operation (2 points), and delayed function that required more than one dialysis procedure (3 points). Acute rejection was defined by the need for treatment, with or without biopsy confirmation. Patients, enrolled to this study, were followed for 2 years until return to dialysis or until December, 2014. In the course of follow-up period GFR was estimated annually.

During the 2nd year of follow-up four grafts failed. For four patients, who returned to dialysis, we imputed a GFR of 10 mL/min/1.73 m^2^. The annualized change (slope) in eGFR (ml/min/1.73 m^2^/year) over a period of 2 years was calculated for each patient, having three eGFR values, by the linear mixed effects model with varying intercept and slope. We determined the proportion of patients having an eGFR drop of ≥ 25% from baseline after 1 and 2 years of follow-up and the proportion of patients with rapid decline in eGFR of > − 5 mL/min/1.73 m^2^/year, since both these measures indicate progressive loss of kidney function [19]. We also calculated the frequency of patients who showed improvement in GFR (increase in eGFR ≥ 25% from baseline). The outcomes measured in this study were the decline in eGFR of > − 5 mL/min/1.73 m^2^/year, and the drop in eGFR of ≥ 25% from baseline after 1 and 2 years of follow-up.

### Statistics

Normally distributed data are expressed as mean ± SD; the results were compared with the Student’s t-test for dependent samples; Pearson’s coefficient of correlation (r) was determined where appropriate. Continuous nonparametric data are expressed as the median (interquartile range); the Spearman’s correlation coefficient (rho) was calculated. Frequency data are expressed as percentages. To identify predictors of eGFR during follow-up, linear regression was used. Variables that had non-Gaussian distribution were natural log-transformed. To identify independent predictors of a certain drop in eGFR after 1 and 2 years of follow-up (≥ 25% from baseline [19]) and rapid decline in eGFR of > − 5 mL/min/1.73 m^2^/year [19] we used univariate logistic regression analysis. The area under the receiver-operator characteristic curve (AUC) was then calculated to assess the capability of each variable considered significant by simple logistic regression to discriminate patients with a drop in eGFR ≥ 25% after 1 and 2 years of follow-up, and patients with rapid decline in eGFR of > − 5 mL/min/1.73 m^2^/year from those who did not meet these criteria. We used logistic regression models to estimate combinations of interleukins and other variables of interest and evaluated the discriminatory ability of the combinations with the AUC. Statistica 7.0 (StatSoft Inc., Tulsa, USA), SPSS (version 19.0 SPSS Inc., Chicago, USA) and Medcalc V.14.8.1 (MedCalc Software bvba, Ostend, Belgium) packages were used for statistical analyses. Statistical significance was assumed for p < 0.05.

## Results

### Baseline characteristics of patients

According to the definition of chronic kidney disease [19] we categorized transplant patients into “normal GFR” and “low GFR” subsets defined as eGFR ≥ 60 mL/min/1.73 m^2^ and < 60 mL/min/1.73 m^2^. The demographics and clinical characteristics of patients are presented in Table 1. Most patients in both subsets received a kidney from a deceased donor and they were on cyclosporine-based immunosuppression. No significant differences in sex, age, type of immunosuppressive drug regimen, time after transplant, frequency of impairment of initial graft function, or acute rejection were observed between groups. The mean arterial pressure and the percentage of patients regularly receiving antihypertensive therapy were significantly higher in the low GFR group. The differences between the main laboratory parameters of graft status (e.g., serum creatinine, eGFR, normalized proteinuria) were highly significant (Table 1).

**Table 1:** Baseline characteristics of the study population

Parameter	Patient groups					
	eGFR at baseline			eGFR decline by 25% at 2 years		
	Normal eGFR, ≥ 60 mL/min/1.73 m^2^ (N = 7)	Low eGFR, < 60 mL/min/1.73 m^2^ (N = 23)	p	No (N = 23)	Yes (N = 7)	p
Recipient age, years	36 (16–47)^a^	38 (29–42)	0.565	36 (24–42)	38 (27–42)	0.962
Recipient gender, males, n (%)	2 (28.6)	14 (60.9)	0.134	13 (56.5)	3 (42.9)	0.526
Calcineurin inhibitor, cyclosporine A use, n (%)	5 (71.4)	13 (56.5)	0.481	15 (65.2)	3 (42.9)	0.290
Antiproliferative agent, mycophenolate mofetil use, n (%)	6 (85.7)	23 (100)	0.065	22 (95.7)	7 (100)	0.575
Anti-IL-2 receptor antibodies use, n (%)	5 (71.4)	15 (65.2)	0.760	17 (73.9)	3 (42.9)	0.127
Type of donor, deceased, n (%)	4 (57.1)	18 (78.3)	0.269	16 (69.6)	6 (85.7)	0.398
Impaired initial function, n (%)	1 (14.3)	6 (26.1)	0.518	4 (17.4)	3 (42.9)	0.163
Previous AR, n (%)	0	2 (8.7)	0.419	1 (4.4)	1 (14.3)	0.356
Time after transplantation (months)	68 (45–128)	68 (41–102)	0.848	68 (36–84)	126 (52–156)	0.096
Serum creatinine (µmol/L)	100 (78–121)	192 (134–247)	< 0.001	136 (115–201)	202 (137–247)	0.077
eGFR (mL/min/1.73 m^2^)	79 (67–91)	34 (31–46)	< 0.001	41 (32–58)	34 (15–67)	0.266
Serum urea (mmol/L)	5.8 (5.2–6.4)	10.4 (8.4–12.7)	< 0.001	9.3 (6.8–12.4)	9.9 (6.1–20.3)	0.632
Proteinuria/creatinine (mg/mmol)	2.7 (0.9–3.6)	9.4 (4.0–20.5)	0.008	6.0 (1.5–10.8)	20.5 (3.3–60.7)	0.029
Proteinuria/creatinine > 15 mg/mmol, n (%)	0	9 (39.1)	0.048	4 (17.4)	5 (71.4)	0.006
Diuresis (L/day)	1.4 (1.1–1.6)	1.4 (1.2–1.7)	0.811	1.4 (1.1–1.5)	1.6 (1.4–1.7)	0.033
Mean arterial pressure (mm Hg)	93 (93–103)	110 (103–116)	0.001	103 (93–116)	116 (103–116)	0.288
Treated hypertension, n (%)	1 (14.3)	14 (60.9)	0.031	10 (43.5)	5 (71.4)	0.195

eGFR, estimated glomerular filtration rate; IL-2, interleukin 2; AR, acute rejection

^a^Median (interquartile range)

### Interleukins’ levels at baseline, their correlation with clinical variables and between each other

Levels of serum and urinary interleukins in patients with normal GFR and low GFR are shown in Table 2. Level of IL-2 in urine was significantly higher in patients with low GFR, whereas levels of urinary IL-8 and serum IL-10 were higher in that subset of patients at the level of trend. Correlations of interleukins concentration with patients’ characteristics at enrollment are shown in Table 3. Level of IL-2 in urine correlated positively with proteinuria, serum creatinine and urea, and it correlated negatively with eGFR. Serum IL-8 correlated positively with deceased donor status, and it correlated negatively with eGFR at the level of trend. Urinary IL-8 correlated positively with serum creatinine and proteinuria. Serum IL-10 correlated negatively with patients’ age and with time after transplantation and correlated positively with serum urea at the level of trend.

**Table 2:** Serum and urinary levels of interleukins at baseline

Interleukins level	Patient groups					
	eGFR at baseline			eGFR decline by 25% at 2 years		
	Normal eGFR, ≥ 60 mL/min/1.73 m^2^ (N = 7)	Low eGFR, < 60 mL/min/1.73 m^2^ (N = 23)	p	No (N = 23)	Yes (N = 7)	p
IL-8 (pg/mL)	3.00 (1.16–3.87)	3.10 (2.28–5.10)	0.501	3.00 (2.19–3.78)	3.87 (2.30–6.85)	0.360
IL-10 (pg/mL)	2.24 (1.49–3.33)	3.53 (2.24–4.48)	0.061	3.30 (2.24–4.01)	3.87 (1.29–4.55)	0.962
IL-2 (pg/mL)	0.20 (0.16–3.19)	0.20 (0.18–2.34)	0.924	0.64 (0.19–3.19)	0.18 (0.18–0.20)	0.107
IL-8/Cr (pg/mL/mmol/L)	0.21 (0.10–0.39)	0.60 (0.31–2.29)	0.077	0.38 (0.10–0.89)	1.18 (0.21–3.31)	0.190
IL-10/Cr (pg/mL/mmol/L)	0.08 (0.05–0.09)	0.10 (0.07–0.22)	0.118	0.09 (0.07–0.21)	0.08 (0.05–0.17)	0.501
IL-2/Cr (pg/mL/mmol/L)	0.15 (0.06–0.22)	0.25 (0.14–0.74)	0.048	0.20 (0.13–0.36)	0.56 (0.13–2.47)	0.159

IL-2, interleukin 2; IL-8, interleukin 8; IL-10, interleukin 10; Cr, creatinine

**Table 3:** Correlation of serum and urinary interleukins with patients’ characteristics at enrollment

Parameter	IL-2	IL-2/Cr	IL-8	IL-8/Cr	IL-10	IL-10/Cr
Recipient age at baseline	–	–	–	–	− 0.425^a^ 0.021^b^	–
Recipient gender	–	–	–	–	–	–
Deceased donor	–	–	0.480 0.018	–	–	–
Impaired initial function	–	–	0.319 0.129	–	–	–
Previous acute rejection	–	–	–	–	–	–
Time post-transplant	–	–	–	–	− 0.463 0.012	–
Serum creatinine	–	0.444 0.016	–	0.409 0.028	–	–
eGFR at baseline	–	− 0.368 0.049	− 0.396 0.055	− 0.321 0.089	− 0.301 0.112	–
Serum urea	0.323 0.088	0.436 0.019	0.371 0.074	0.341 0.070	0.365 0.052	–
Proteinuria/Cr	–	0.705 < 0.001	–	0.371 0.047	–	0.338 0.072
Diuresis	–	–	–	–	–	–
Mean arterial pressure	–	0.340 0.071	0.349 0.095	0.316 0.095	–	–

–, none or weak correlation (rho < 0.3); IL-2, interleukin 2; IL-8, interleukin 8; IL-10, interleukin 10; Cr, creatinine; eGFR, estimated glomerular filtration rate

^a^Spearman’s correlation coefficient (rho)

^b^Level of statistical significance (p)

Table 4 provides details of correlations between interleukins. We observed no correlation between interleukins in serum. We also did not find correlations between any interleukin in serum and its counterpart in urine. Concentrations of IL-2 and IL-8 in urine correlated between each other. We found correlation between IL-10 in serum and IL-8 in urine.

**Table 4:** Internal correlations between levels of interleukins at enrollment

	IL-2	IL-2/Cr	IL-8	IL-8/Cr	IL-10	IL-10/Cr
IL-2		0.259^a^ 0.175^b^	− 0.156 0.467	0.027 0.889	0.002 0.992	0.248 0.194
IL-2/Cr			− 0.095 0.659	0.468 0.011	0.002 0.992	− 0.065 0.738
IL-8				0.150 0.483	0.255 0.229	− 0.074 0.730
IL-8/Cr					0.392 0.035	0.351 0.062
IL-10						− 0.001 0.994

IL-2, interleukin 2; IL-8, interleukin 8; IL-10, interleukin 10; Cr, creatinine

^a^Spearman’s correlation coefficient (rho)

^b^Level of statistical significance (p)

### The evolution of allograft function and the predictive variables

Associations of clinical and laboratory variables with eGFR during follow-up are presented in Table 5. Apart from several clinical variables, both IL-2 in urine and IL-8 in serum were significantly associated with eGFR after 1 and 2 years of follow-up, whereas serum IL-10 demonstrated weak correlation only with eGFR after 1 year of follow-up.

**Table 5:** Significant predictors of eGFR after one and 2 years of follow-up

Predictive variables	eGFR after 1 year of follow-up	eGFR after 2 years of follow-up
Time post-transplant		− 0.369 (0.176), p = 0.045
Ln mean arterial pressure^c^	− 0.645^a^ (0.144)^b^, p < 0.001	− 0.619 (0.148), p < 0.001
eGFR at baseline	0.786 (0.117), p < 0.001	0.733 (0.129), p < 0.001
Ln proteinuria/Cr^c^	− 0.657 (0.143), p < 0.001	− 0.674 (0.140), p < 0.001
Ln IL-2/Cr^c^	− 0.481 (0.169), p = 0.008	− 0.502 (0.166), p = 0.006
Ln IL-8^c^	− 0.616 (0.172), p = 0.002	− 0.594 (0.176), p = 0.003
Ln IL-10^c^	− 0.393 (0.177), p = 0.035	

Only variables that significantly influenced the eGFR in univariate analysis are included

eGFR, estimated glomerular filtration rate; IL-2, interleukin 2; IL-8, interleukin 8; IL-10, interleukin 10; Cr, creatinine; p, level of statistical significance

^a^Standardized regression coefficient (beta)

^b^Standard error of beta

^c^Natural log-transformed variables

After 1 year of follow-up, the mean eGFR did not change significantly in the total patient population: 44.2 ± 21.6 mL/min/1.73 m^2^ compared to eGFR at baseline 45.8 ± 21.5 mL/min/1.73 m^2^ (p = 0.540). Four patients (17.4%) from the low GFR group and one patient (14.3%) from the normal GFR group (p = 0.847) demonstrated a decline in eGFR ≥ 25% from baseline after 1 year due to chronic allograft dysfunction, without histological confirmation.

During the second year of follow-up a total of four grafts failed. The cause of allograft failure was verified histologically in one case and we found chronic active antibody-mediated rejection. After 2 years of follow-up the mean eGFR did not decrease significantly (42.0 ± 21.8 mL/min/1.73 m^2^) compared with that at baseline (p = 0.199). Nevertheless, this decline was significant compared to eGFR after 1 year (p = 0.006). Five patients, who exhibited decline in eGFR by ≥ 25% at 1 year, still displayed it at 2 years. Two more patients from normal and low GFR subsets demonstrated drop in eGFR ≥ 25%. Overall five patients (21.7%) from the low GFR group and two patients (28.6%) from the normal GFR group had a decrease in eGFR ≥ 25% compared with baseline (p = 0.708). We did not observe any new intercurrent events during the second year of follow-up. Three patients (13%) from the low GFR group showed an increase in eGFR ≥ 25% after 1 year and still displayed it after 2 years, whereas such patients were absent in the normal GFR group (p = 0.314). The presumed causes of improving graft function were conversion of immunosuppression from cyclosporine A to tacrolimus in two patients and effective antibiotic treatment of urinary tract infection in one patient.

Frequency of significant proteinuria and levels of median proteinuria at baseline were higher in patients with the eGFR decline by ≥ 25% after 2 years (Table 1) and after 1 year (data not shown) of follow-up. Those patients had higher diuresis, as well. Median levels of serum and urinary interleukins at baseline were not significantly different between patients who lost 25% of GFR by the end of follow up and those who did not. However, median normalized urinary IL-2 level at baseline was higher at the level of trend in patients who showed a decrease in GFR by ≥ 25% after 1 year of follow-up (N = 5) compared to those who did not: 0.738 (0.346–2.465) pg/mL/mmol/L vs. 0.200 (0.128–0.361) pg/mL/mmol/L, p = 0.065.

Table 6 summarizes the logistic regression for predictors of the eGFR decline by ≥ 25% after 1 and 2 years of follow-up. Higher normalized proteinuria and higher urinary IL-2 concentrations predicted the decline in eGFR of ≥ 25% after 1 year of follow-up. With respect to the graft function after 2 years of follow-up higher normalized proteinuria and longer time post-transplant were significantly associated with higher odds ratio of certain eGFR drop (Table 6). The discriminating ability of each variable, deemed significant by simple logistic regression, for the drop in eGFR ≥ 25% after 1 and 2 years of follow-up was determined by the AUC analysis. As illustrated in Table 7, normalized proteinuria predicted significant decline in eGFR by ≥ 25% after 1 year with good power, whereas urinary IL-2 exhibited only fair accuracy. Nevertheless, the AUCs for these two variables were not significantly different from each other (p > 0.05). The combination of normalized proteinuria with urinary IL-2 resulted in an AUC of 0.858 (Table 7), which indicated good power as well. This AUC showed slightly higher predictive power than AUCs for normalized proteinuria and urinary IL-2 separately, but without statistical significance. Proteinuria and time after transplantation both exhibited fair discriminatory power in predicting the eGFR decline by ≥ 25% after 2 years of follow-up (Table 7), without significant differences between AUCs (p > 0.05). The combination of proteinuria and time after transplantation yielded the AUC of 0.799, which was higher than the AUC for each predictor alone, but not significantly (p > 0.05).

**Table 6:** Significant predictors of eGFR decline by ≥ 25% during follow-up

Predictive variables	Univariate logistic regression		
	OR	CI	p-level
eGFR decline by ≥ 25% after 1 year of follow-up, N = 5			
Proteinuria/Cr	3.08	1.02–9.28	0.030
IL-2/Cr	2.94	1.06–8.18	0.038
eGFR decline by ≥ 25% after 2 years of follow-up, N = 7			
Proteinuria/Cr	2.45	1.02–5.92	0.046
Time post-transplant	1.02	1.00–1.05	0.038

Only variables that significantly influenced the eGFR decline in univariate analysis are included

eGFR, estimated glomerular filtration rate; OR, odds ratio; CI, 95%-confidence interval; IL-2, interleukin 2; Cr, creatinine; N, number of patients with certain decline in eGFR

**Table 7:** AUC for potential markers of eGFR decline by ≥ 25% during follow-up

Predictive variables	AUC	CI	p-level
eGFR decline by 25% after 1 year of follow-up, N = 5			
Proteinuria/Cr	0.817	0.584–1.000	0.028
IL-2/Cr	0.767	0.519–1.000	0.065
Proteinuria/Cr + IL-2/Cr	0.858	0.670–1.000	0.013
eGFR decline by 25% after 2 years of follow-up, N = 7			
Proteinuria/Cr	0.779	0.561–0.997	0.028
Time post-transplant	0.705	0.451–0.958	0.108
Proteinuria/Cr + Time post-transplant	0.799	0.597–1.000	0.019

Only variables that significantly influenced the eGFR decline in univariate logistic regression are included

AUC, areas under the receiver operating characteristic curve; CI, 95%-confidence interval; eGFR, estimated glomerular filtration rate; IL-2, interleukin 2; Cr, creatinine; N, number of patients with certain decline in eGFR

The mean eGFR slope in the overall group of patients was − 1.9 ± 8.1 mL/min/1.73 m^2^/year (median: − 1.0 mL/min/1.73 m^2^/year). In the normal GFR group, the slope was − 4.0 (− 10.0 to − 1.5) mL/min/1.73 m^2^/year, which was higher compared to the low GFR group at the level of trend: 0.5 (− 3.0 to 4.5) mL/min/1.73 m^2^/year (p = 0.054). Three patients (13.0%) in the low GFR group and three patients (42.9%) in the normal GFR group lost GFR at a rate of > − 5.0 mL/min/1.73 m^2^/year (p = 0.084). All of them got into the group of seven patients with the decrease in eGFR by ≥ 25% at 2 years. Among all tested variables, only time after transplantation significantly predicted the rapid decline in eGFR by > − 5 mL/min/1.73 m^2^/year in univariate logistic regression [odds ratio (OR) = 1.03, confidence interval (CI) 1.00–1.06, and p = 0.020], whereas the effect of IL-2 was only at the level of trend (OR = 2.14, CI 0.88–5.22, and p = 0.079). The discriminating ability of time post-transplant and IL-2 for the drop in eGFR by > − 5 mL/min/1.73 m^2^/year was determined by the AUC analysis. Both time post-transplant and urinary IL-2 predicted rapid decline in eGFR by > − 5 mL/min/1.73 m^2^/year with fair power (AUC: 0.779, CI 0.528–1.000, p = 0.038, and AUC: 0.703, CI 0.441–0.964, p = 0.132, respectively), although the effect of IL-2 was not significant. The AUCs for time post-transplant and IL-2 did not differ significantly (p > 0.05). The combination of time post-transplant with urinary IL-2 resulted in the AUC of 0.855 (CI 0.687–1.000, p = 0.008) in predicting rapid decline of eGFR, which indicated a good power, but was not significantly different from the AUC for time post-transplant alone.

## Discussion

There is a strong medical rationale for the development of new diagnostic and predictive noninvasive biomarkers for KAG status. This paper explored the question of whether serum and urinary IL-2, IL-8 and IL-10 in the late postoperative period could predict a decline in GFR over a follow-up period of 2 years. The principal finding of our study is the association between higher urinary levels of IL-2 in the late post-transplant period with declining kidney allograft function. Higher urinary IL-2 concentrations were not only associated with lower eGFR at baseline and during 2 years of follow-up, but also predicted a certain drop in eGFR of ≥ 25% after 1 year of follow-up. With respect to discriminative characteristics the combination of time after transplantation and urinary IL-2 predicted rapid decline in eGFR by > − 5 mL/min/1.73 m^2^/year with good accuracy. We also established that higher serum concentrations of IL-8 were associated with lower eGFR after 1 and 2 years of follow-up, whereas higher serum concentrations of IL-10 demonstrated significant association only with lower eGFR after 1 year.

As higher urinary IL-2 concentrations are associated with lower eGFR, higher serum creatinine and urea, and higher proteinuria at baseline, we believe that IL-2 plays role in the pathogenesis of late renal allograft dysfunction. Considering activated T-cells as the main source of IL-2 [5] and a certain role of IL-2 in graft pathology [6, 12, 13] we hypothesize that high urinary IL-2 reflects ongoing T-cell-dependent alloimmune response in late post-transplant period, which is supported by the literature [11, 20]. Apart from the stimulation of T-cell-mediated alloimmunity IL-2 also drives effector B cells development [5], which might occur within the graft itself [21], and thus it could contribute to chronic antibody-mediated rejection. Antibody-mediated rejection itself is recognized as a leading cause of late kidney allograft dysfunction [22] and it often coincides with T-cell-mediated rejection [22]. Another possible explanation for the relationship between high urinary IL-2 concentrations with KAG dysfunction is an underlying autoimmune process, e.g. some types of glomerulonephritis, as Chan et al. showed high IL-2 renal gene expression in lupus nephritis [23]. Furthermore, IL-2 could itself induce apoptosis of tubular epithelial cells [24], thereby worsening allograft function. It is also worth considering the possibility that high urinary IL-2 reflects a non-specific inflammation in the grafts. This is supported by the correlation between urinary concentrations of IL-2 and proinflammatory IL-8 [5]. Correlations between urinary IL-8 and proteinuria and serum creatinine, and between serum IL-8 with eGFR at all time points also argue in favor of this hypothesis. Literature data suggest an implication of IL-8 in KAG dysfunction associated with urinary tract infections [8, 9, 16]. Budde et al. observed elevated urinary IL-8 in patients with acute graft rejection [9]. Non-specific secondary inflammation in the allograft is considered to be a part of an active injury–repair response [22], which is the strongest correlate of subsequent functional disturbance of the graft and progression to failure. Our results also indirectly support the feasibility of using anti-IL-2 therapy not only for the induction immunosuppressive therapy, but also for prevention and treatment of acute rejection in post-transplant period [25]. A correlation between serum IL-10 and eGFR after 1 year of follow-up might be attributed to a role of IL-10 in initiation and/or maintenance of chronic allograft rejection [11], because IL-10 serves as a growth and differentiation factor for B cells [5] and it might be also involved in the development and progression of kidney fibrosis [5, 18].

Seventeen and twenty-three percent of patients met criteria of certain decline in eGFR after 1 and 2 years, respectively, and twenty percent of patients displayed rapid decline in eGFR of − 5 mL/min/1.73 m^2^/year. Although median urinary IL-2 level did not differ significantly between patient subgroups with declining and stable function, it significantly predicted a drop in eGFR by ≥ 25% after 1 year of follow-up. For a one-unit increase in urinary IL-2 concentration, the odds ratio for certain drop in eGFR was 2.94. However, with respect to discriminative characteristics, urinary IL-2 only slightly improved predictive value of proteinuria for certain decline in eGFR after 1 year. Loss of graft function did not depend on GFR at baseline what was reported earlier [2]. In this regard urinary IL-2, which provides information about additional injuries to the graft, might improve prediction. Significant effect of IL-2 on the drop in eGFR does not expand beyond 1 year, which might indicate abatement of pathological process, mediated by IL-2. Our hypothesis is in line with the findings that T-cell-mediated lesions in kidney graft were not associated with increased risk of graft failure [22].

The longer time post-transplant significantly predicted certain decline in eGFR after 2 years of follow-up in logistic regression, and it also predicted rapid decline in eGFR by > − 5 mL/min/1.73 m^2^/year with fair accuracy. These effects can be explained by larger cumulative injuries with increasing time [22]. We also found that IL-2 further improved performance of time after transplantation in predicting rapid decline of KAG function since their combination had higher prognostic accuracy than that for the time after transplantation alone. Although this effect is not significant, it is confirmed by the shift of AUC from a range of a fair accuracy to a good one [26]. Though it is difficult to make any conclusions on so few patients, taken together, these data suggest that high urinary level of IL-2 in late post-transplant period reflects ongoing antigen-specific immune response or non-specific inflammatory process in the graft, which may lead to decline of eGFR in the near future. Morphological correlates of failing renal graft are glomerulosclerosis, tubular atrophy and interstitial fibrosis [3, 22]. According to several reports, if interstitial fibrosis is accompanied by inflammation, graft function deteriorates more rapidly [3]. IL-2, secreted mostly by T cells in the graft, can interact with epithelial cells, leading to a decrease in type IV collagen production in these cells, thus contributing directly to tubular atrophy [27]. IL-2 can also indirectly induce fibroblasts by stimulating interstitial macrophages [18, 27]. Our results are in line with the concept that progression of KAG dysfunction can be explained only as a consequence of ongoing disease or injury in the graft [22]. The obtained results support a need for further research to assess the diagnostic and prognostic value of urinary IL-2 measurements in renal allograft diseases.

We also identified proteinuria in the late post-transplant period as a predictor of certain decline of allograft function after 1 and 2 years of follow-up. Negative impact of proteinuria on KAG function and survival is well-recognized [3, 28, 29]. Proteinuria in our patients correlated with both urinary IL-2 and IL-8 that points to association of proteinuria with ongoing injury in the KAG. It is known that proteinuria itself can cause injury of tubular epithelial cells and stimulate them to the synthesis of chemokines [29], which can contribute to inflammation in tubulointerstitium in native [29] as well as in transplanted kidneys [28]. Thus, we speculate that tubular damage, induced by proteinuria, can induce or potentiate immunological processes in the graft, which display themselves as an increase in urinary IL-2 levels; however, this hypothesis of course needs to be further proved. Our results suggest some more clinical implications. Measurements of urinary IL-2 might help elucidate the processes leading to the graft failure. Urinary IL-2 seems to be superior to serum IL-2 as well as to IL-8 and IL-10 in serum and urine in differentiating KAG recipients who subsequently will lose KAG function from those who will not. IL-2 provides an accurate and quick measurement by a commonly available cost-efficient ELISA, which can readily be implemented in clinical labs. Association of elevated urinary IL-2 with both low eGFR and decline in eGFR from baseline might have considerable clinical impact, since these surrogates are directly related to inferior allograft survival [2, 19]. Thereby, our findings expand results of previous studies on the predictive variables of GFR drop. Another interesting observation is the absence of correlation between different interleukins in serum, and between any interleukin in the serum and its counterpart in urine. Altogether, our findings allow us to think that urinary interleukins have renal origin and should be regarded as more reliable markers of KAG status.

This study is limited by several factors. It was a single-center study carried out on a small group of Caucasian patients and restricted to only 2 years of follow-up period. So our conclusions have only preliminary character and should be replicated in a longer study with a larger cohort representative of the general population of kidney transplant recipients. We used a creatinine-based measure of GFR rather than a gold standard measurement of GFR, what, however, is allowable in accordance with the recommendations of recent guidelines [19]. The creatinine measurements were not calibrated with respect to a reference laboratory. However, because all creatinines were measured in the same laboratory, possible calibration error cannot explain the observed differences in eGFR values. The next limitation is that GFR estimation was not paired with measurement of interleukins at all time points. These data could help elucidate the processes leading to graft failure. At last, we can only assume the etiology of dysfunction because allograft biopsy was performed only in individual patients. It is possible that future combination of urinary IL-2 measurement with biopsy results will improve diagnostics and prediction of kidney transplant pathology.

## Conclusions

Overall, our study has revealed the association between higher urinary levels of IL-2 in the late post-transplant period with declining kidney allograft function. Higher urinary IL-2 concentrations were associated with lower eGFR at baseline and during the first and the second year of follow-up. Higher IL-2 in urine also predicted decline in eGFR of ≥ 25% after 1 year of follow-up. When combined with time after transplantation, urinary IL-2 demonstrated good accuracy in predicting rapid decline in eGFR by > − 5 mL/min/1.73 m^2^/year. Our findings suggest that urinary IL-2 in the late period after kidney transplantation have promise in identifying patients who are at risk for progressive loss of graft function and might benefit from closer monitoring and intervention.

## Abbreviations

KAG: kidney allograft
IL: interleukin
eGFR: estimated glomerular filtration rate
AUC: area under the receiver-operator characteristic curve
OR: odds ratio
CI: confidence interval
